# Forensic autopsy costs in the city of São Paulo

**DOI:** 10.1590/S1516-31802003000300011

**Published:** 2003-05-01

**Authors:** Fernando Augusto Mardiros Herbella, Pedro Herbella Fernandes, Carlos Delmonte, José Carlos Del Grande

**Keywords:** Autopsy, Forensics, Costs analysis, Health economy, Autópsias, Forense, Análise de custos, Economia da saúde

## Abstract

**CONTEXT:**

Modern medical practice involves cost analysis of therapeutic and diagnostic procedures. There are no papers dealing with this theme in relation to forensic autopsies in our country.

**OBJECTIVE:**

Analysis of direct costs of forensic autopsies.

**TYPE OF STUDY:**

Cost analysis.

**SETTING:**

São Paulo Medical Examiner's Central Office.

**SAMPLE:**

Year 2001 activity.

**PROCEDURES:**

Routine forensic autopsies.

**MEAN MEASUREMENTS:**

Analysis of direct costs of personnel and material.

**RESULTS:**

Cost of personnel represents 90.38% or US$ 93.46. Material expenses comprised 9.62% or US$ 9.95. Total costs were calculated to be US$ 103.41.

**CONCLUSIONS:**

Forensic autopsies have a high cost. Cases to be autopsied should be judiciously selected. Our results are similar to international studies if data are rearranged based on the number of annual necropsies.

## RESUMO

**CONTEXTO:**

A medicina moderna preocupa-se com análise de custos para os procedimentos diagnósticos e terapêuticos, não havendo trabalhos pertinentes a autópsias em nosso meio.

**OBJETIVO:**

Avaliar os custos diretos de uma necropsia forense.

**LOCAL:**

Instituto Médico Legal Sede de São Paulo.

**AMOSTRA:**

Movimento do ano de 2001.

**PROCEDIMENTOS:**

Autópsias forenses de rotina.

**VARIÁVEIS ESTUDADAS:**

Análise de despesas com recursos humanos e material consumível na realização de necropsias forenses.

**RESULTADOS:**

Os custos com recursos humanos corresponderam a US$ 93,46 ou 90,38% dos custos totais, cabendo às despesas materiais US$ 9,95 ou 9,62%. O custo total foi estimado em US$ 103,41 por procedimento.

**CONCLUSÕES:**

O exame necroscópico forense apresenta alto custo para o Estado devendo haver critério nos casos a serem necropsiados, corrigindo-se os custos de acordo com o volume de necropsias. Nossos resultados são semelhantes aos de trabalhos internacionais se os dados forem reorganizados com base no número anual de necropsias.

## INTRODUCTION

Cost analysis of therapeutic and diagnostic procedures, for medical and nonmedical reasons, is a growing interest among researchers in all specialties.^1-3^ However, few papers have dealt with the subject in relation to autopsies.^4,6^ In our country, where the number of forensic autopsies is rising due to increasing urban violence, there are no studies on the subject.

## METHODS

The authors evaluated the direct costs of forensic autopsies at the São Paulo Medical Examiner 's Central Office (Instituto Médico Legal). The São Paulo Medical Examiner's Central Office works continuously, 24 hours a day, 365 days a year. It is responsible for cadavers coming from the central and northern areas of the city, as well as special cases (priorities, putrefaction, burns, prisoners etc.)

Indirect costs, such as those linked to acquisition, maintenance, conservation, depreciation and cleaning of permanent equipment and facilities were not considered. The Purchasing Division of the Technical-Scientific Police Superintendency of São Paulo provided expenditure data related to the acquisition of non-permanent materials.

Expenditure data related to personnel were calculated on the basis of multiplying the annual salary (fringe benefits included) of a medium-category worker (class 3) and the total number of workers in that category, and then dividing by the number of autopsies done in a year. Most of the employees are part-time workers.

The American dollar was adopted as the currency (rate of exchange as provided by Central Bank of Brazil, May 15, 2002, of R $2.50/dollar).

## RESULTS

In the most recent year for which statistics are available (2001), 6,244 autopsies were done at the São Paulo Medical Examiner's Central Office.

### Cadaver transportation

Cadaver transportation requires a staff of 3 teams of 2 drivers (cost for each case US$ 4.05) and 3 teams of 2 morgue attendants (cost for each case US$ 4.05) (Table 1).

**Table 1:** Personnel costs

Profession	Number of workers per team	Number of teams	Annual salary (US$) (individual)	Cost of each autopsy (US$)
Administrative staff	2	3	3100.36	2.98
Driver	2	3	4210.37	4.05
Fingerprint examiner	1	7	6092.84	6.83
Photographer	1	7	6092.84	6.83
Morgue attendant	4	3	4210.37	8.09
Secretary	5	2	5797.71	9.28
Autopsy technician	4	3	6092.84	11.71
Medical examiner	4	5	13640.82	43.69
	**Total**			**93.46**

Supply costs include, for each case: 2 pairs of gloves (US$ 0.72), 1 cadaver bag (US$ 4.07), 1 identification tag (US$ 0.05), and 1 registration sheet (US$ 0.01).

Vehicle expenses were calculated with respect to the mean fuel consumption, i.e. 41.5 liters of diesel for each vehicle per day or 4.8 liters per case (US$ 1.68). Each vehicle covers a mean of 217.27 kilometers per day.

The total expenditure on cadaver transportation and handling is US$ 14.63.

### Identification and registration

As part of the cadaver identification, fingerprints are routinely taken. Costs include fees for fingerprint examiners (7 teams of 1 examiner, costing US$ 6.83) and 1 sheet for fingerprint printing (US$ 0.01).

Photographs are taken digitally and then stored. They are not printed unless requested by the police. Fees for the photographer (7 teams of 1 photographer) account for US$ 6.83. A computer disk is used for storage (US$ 0.40).

The total cost for identification and registration is US$ 14.07.

### Administrative work

The administrative work encompasses receiving the police examination requisition, registering the death certificate and informing the families. It requires a staff of 3 teams of 2 administrative workers (US$ 2.98), 1 sheet (US$ 0.01), 1 facsimile sheet (US$ 0.01) and 1 local telephone call (US$ 0.03).

Total cost for administrative work is US $ 3.03.

### Autopsy

The autopsy room employees 5 teams of 4 medical examiners (US$ 43.69), 3 teams of 4 autopsy technicians (US$ 11.71), and 3 teams of 2 morgue attendants (US$ 4.05). Supplies used are: 2 pairs of gloves (US$ 0.72), 10 liters of water (US$ 0.13), and 1 meter of suture string (US$ 0.01).

Total cost for the autopsy is US$ 60.31.

### Autopsy report

The autopsy report uses, from the manuscript to the typed final form, 7 sheets (US$ 0.07) and employees 2 teams of 5 secretaries (US$ 9.28).

Total cost for the autopsy report is US$ 9.35.

### Total Costs

The total direct cost for one autopsy is US $ 103.41.

**Figure 1:**
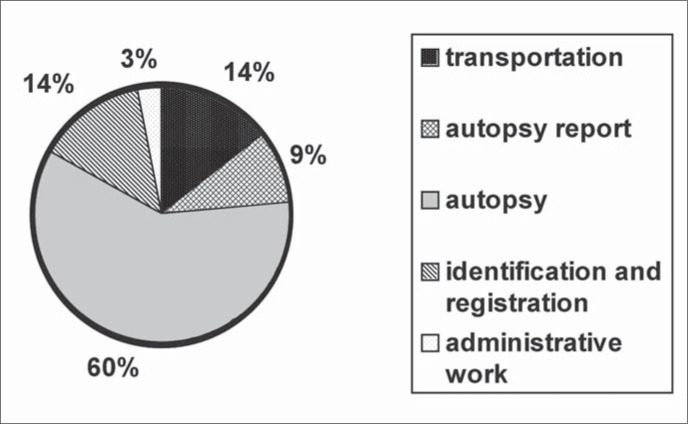
Cost according to each part of the autopsy process.

Figure 1 shows the estimated cost for each part of the process.

### Personnel

**Figure 2:**
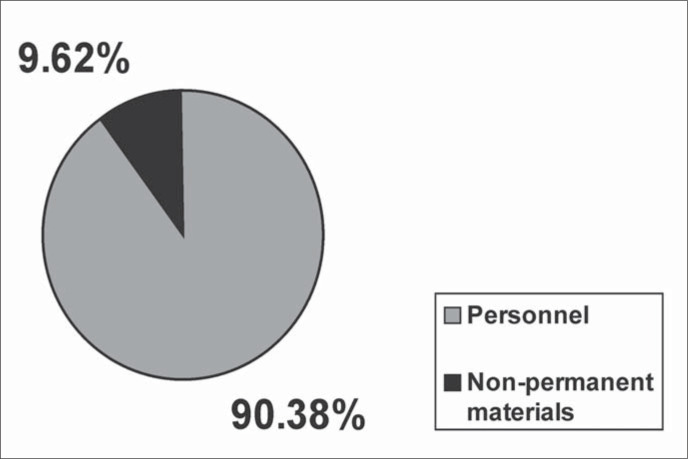
Personnel and non-permanent materials costs.

Staff salaries account for US$ 93.46 (Table 1) or 90.38% of the expenditure (Figure 2).

### Supplies

Non-permanent materials account for US$ 9.95 (Table 1) or 9.62% of the expenditure (Figure 2).

## DISCUSSION

The scientific value of autopsies is undeniable. However, international data show a decline in the number of autopsies, and economic reasons are a possible factor.^7^ In our country, the number of forensic autopsies is rising due to increasing urban violence. Data from the Program for Improvements in Death Cause Information of São Paulo (PRO-AIM)^8^ blame homicide for the second biggest cause of deaths in the city of São Paulo, and the first among men, thus constituting a real public health problem. Obviously, a well-performed forensic autopsy makes an incontestable contribution to the justice system. However, as in other countries, judicious selection of which cases should be autopsied or not would provide economic benefits for the State and better attention for the selected cases.

It is not an easy task to estimate the cost of an autopsy. Simple division of the annual budget for the Instituto Médico Legal of São Paulo by the number of autopsies per year is not feasible, because of the diversity of tasks it performs (traumatology reports, toxicology laboratory, sexological and anthropological examinations etc.), which are not limited to the medical examiner's office.^5^ This is also one of the reasons why indirect costs were not taken into account in this study. Yesner^5^ esti-mated an indirect cost of US$ 170.00 for each autopsy in his service. Obviously, the real cost of an autopsy must be higher than our estimate, because of such indirect costs,^2^ direct costs not added, such as leadership, security, etc, and minimal expenditures^3^ that are impossible to estimate, like pen ink, etc.

There are few previous papers^5-7^ concerning autopsy costs with which comparisons can be made, and none of them is from our country. These papers have reported costs ranging from US$ 800.00 to US$ 2000.00,^5,7^ with variations due to the complexity of the procedures, inclusion of laboratory test, etc. Indirect costs represented approximately 15% of the total cost,^5,7^ personnel salaries 50 to 70%,^5,7^ and supplies 10 to 40%.^5,7^ These proportions are similar to our results if indirect costs are not considered.

Our cost per case seems to be low, in comparison with data from studies in other countries. Nevertheless, the high number of autopsies per year justifies the results. Clearly, the total cost for a service increases as the number of cases increases, although if personnel costs are stable, the cost per autopsy will decrease as the annual number of cases increases.^5^ If our personnel expenditure is kept unchanged and our costs are calculated for 550 autopsies/year, as in the study by Yesner,^5^ our estimated cost would be US$ 1048.04 for each case, which is similar to the cost calculated by that author, i.e. US$ 955.00. In the same way, if Yesner^5^ had had our volume of 6244 autopsies per annum, his estimated cost would have decreased to US$ 158.59.

## CONCLUSION

Forensic autopsies represent a high cost to the State. Judicious criteria should be used for selecting cases for autopsy, like in other countries. Cases in which a crime or violence is not involved- like unknown identification, femur fractures due to osteoporosis, prisoners with natural causes of death, etc. – lead to a loss of money and time that could be better used in selected cases. Our results are similar to studies from other countries if the annual number of autopsies is considered.
